# Can morphological MRI differentiate between primary central nervous system lymphoma and glioblastoma?

**DOI:** 10.1186/s40644-016-0098-9

**Published:** 2016-11-29

**Authors:** H. Malikova, E. Koubska, J. Weichet, J. Klener, A. Rulseh, R. Liscak, Z. Vojtech

**Affiliations:** 1 Department of Radiology, Na Homolce Hospital, Roentgenova 2, Prague, 15000 Czech Republic; 2 Department of Radiology, Third Faculty of Medicine, Charles University in Prague and Faculty Hospital Kralovske Vinohrady, Ruska 87, Prague, 10000 Czech Republic; 3 Department of Neurosurgery, Na Homolce Hospital, Roentgenova 2, Prague, 15000 Czech Republic; 4 Department of Radiology, 1st Faculty of Medicine and General University Hospital, Charles University, Prague, Czech Republic; 5 Department of Stereotactic and Radiation Neurosurgery, Na Homolce Hospital, Roentgenova 2, Prague, 15000 Czech Republic; 6 Department of Neurology, Na Homolce Hospital, Roentgenova 2, Prague, 15000 Czech Republic; 7 Department of Neurology, Third Faculty of Medicine, Charles University in Prague, Ruska 87, Prague, 10000 Czech Republic

**Keywords:** Conventional MRI, Enhancement, Initial evaluation

## Abstract

**Background:**

Primary central nervous system lymphoma (PCNSL) is a rare, aggressive brain neoplasm that accounts for roughly 2-6% of primary brain tumors. In contrast, glioblastoma (GBM) is the most frequent and severe glioma subtype, accounting for approximately 50% of diffuse gliomas. The aim of the present study was to evaluate morphological MRI characteristics in histologically-proven PCNSL and GBM at the time of their initial presentation.

**Methods:**

We retrospectively evaluated standard diagnostic MRI examinations in 54 immunocompetent patients (26 female, 28 male; age 62.6 ± 11.5 years) with histologically-proven PCNSL and 54 GBM subjects (21 female, 33 male; age 59 ± 14 years).

**Results:**

Several significant differences between both infiltrative brain tumors were found. PCNSL lesions enhanced homogenously in 64.8% of cases, while nonhomogeneous enhancement was observed in 98.1% of GBM cases. Necrosis was present in 88.9% of GBM lesions and only 5.6% of PCNSL lesions. PCNSL presented as multiple lesions in 51.9% cases and in 35.2% of GBM cases; however, diffuse infiltrative type of brain involvement was observed only in PCNSL (24.1%). Optic pathways were infiltrated more commonly in PCNSL than in GBM (42.6% vs. 5.6%, respectively, *p* <0.001). Other cranial nerves were affected in 5.6% of PCNSL, and in none of GBM. Signs of bleeding were rare in PCNSL (5.6%) and common in GBM (44.4%); *p* < 0.001. Both supratentorial and infratentorial localization was present only in PCNSL (27.7%). Involvement of the basal ganglia was more common in PCNSL (55.6%) than in GBM (18.5%); (*p* < 0.001). Cerebral cortex was affected significantly more often in GBM (83.3%) than in PCNSL (51.9%); mostly by both enhancing and non-enhancing infiltration.

**Conclusion:**

Routine morphological MRI is capable of differentiating between GBM and PCNSL lesions in many cases at time of initial presentation. A solitary infiltrative supratentorial lesion with nonhomogeneous enhancement and necrosis was typical for GBM. PCNSL presented with multiple lesions that enhanced homogenously or as diffuse infiltrative type of brain involvement, often with basal ganglia and optic pathways affection.

## Background

Although glioblastoma (GBM) and primary central nervous lymphoma (PCNSL) differ in many respects, it is often reported that morphological differentiation between them by MRI is difficult [1]. PCNSL is a rare, aggressive brain neoplasm that may involve the brain, leptomeninges, spinal cord and eyes without systematic lymphomatous involvement, and accounts for approximately 2-6% of primary brain tumors [2]. PCNSL is typically diffuse large B-cell lymphoma (DLBCL), and rarely other types such as Burkitt lymphoma, T cell lymphoma or Hodgkin lymphoma [3, 4]. In immunocompetent patients, PCNSL usually affects older individuals with a slight male predilection and its incidence has been increasing in recent years [5–8]. Immunocompromised patients especially with AIDS have an increased risk of PCNSL, which develops at younger age [5]. The treatment of choice for PCNSL is radiation therapy and/or chemotherapy [9]. In contrast, gliomas are the most common primary brain tumors, accounting for roughly 70% of all primary brain tumors in adults. GBM is the most frequent and severe glioma subtype and accounts for about approximately 50% of diffuse gliomas. It is characterized by infiltration beyond the enhancing margin and with rapid growth [10, 11]. The primary treatment for GBM is surgical resection followed by radiation therapy and chemotherapy [12].

It has been suggested that MRI has limited potential in differentiating between PCNSL and GBM [1], and several studies have employed advanced MRI techniques such as diffusion-weighed imaging (DWI), MR spectroscopy or dynamic contrast enhancement [13–16]. Systematic evaluation of conventional morphological MRI manifestations of both tumors is, however, lacking. Therefore, we endeavored to compare morphological signs on MRI in sufficiently large groups of GBM and PCNSL patients at their initial presentation, thus on the first diagnostic MRI. Only immunocompetent PCNSL subjects were included due to potential confounding effects of pharmacotherapy in immunocompromised patients, such as corticosteroid therapy.

## Methods

### Patient selection

We retrospectively evaluated all available MRI examinations and medical records in patients with histologically proven PCNSL and GBM. All patients provided written, informed consent to treatment and agreed with publishing medical data in scientific literature in anonymous form. This retrospective study was approved by the Ethics committee of Na Homolce Hospital, Prague, Czech Republic.

#### Group A (PCNSL)

Patients with histologically-proven CNS lymphoma acquired at our institution from 2007–2015 were included. DLBCL was histologically proven in all patients, and systemic lymphoma was excluded by bone marrow biopsy, whole-body computed tomography (CT) or whole-body positron emission tomography/CT. From 64 subjects with PCNSL, 54 were immunocompetent while 10 were excluded due to known immunodeficiency. Thus, 54 immunocompetent PCNSL patients were included. Histological specimens were obtained by stereotactic biopsy (*N* = 43), open surgical biopsy (*N* = 6) or by open surgical resection (*N* = 5).

#### Group B (GBM)

Fifty-four consecutive patients with histologically proven GBM (WHO grade IV) acquired at our institution from 2012-2013 were additionally included. Subjects with secondary upgrade of previously known low grade glioma were excluded. The diagnosis was confirmed by histological examination of specimens obtained by open surgery with one exception, in which the specimen was obtained by stereotactic biopsy.

### MRI examination

All MRI examinations were performed at different whole-body 1.5 T scanners and included the following sequences: fast spin echo (FSE) T2-weighted images (T2 WI), T2-weighted fluid-attenuated inversion recovery (FLAIR), susceptibility-weighted images (SWI) or T2* gradient echo (GE). Inconstantly diffusion-weighted images (DWI; b-value 1000) and ADC maps were available (in 35 PCNSL patients and in 51 GBM patients). After intravenous gadolinium contrast administration, spin echo (SE) T1 WI or GE T1 3D were always performed. All MRI examinations were evaluated by consensual reading by 2 experienced radiologists.

**Fig. 1:**
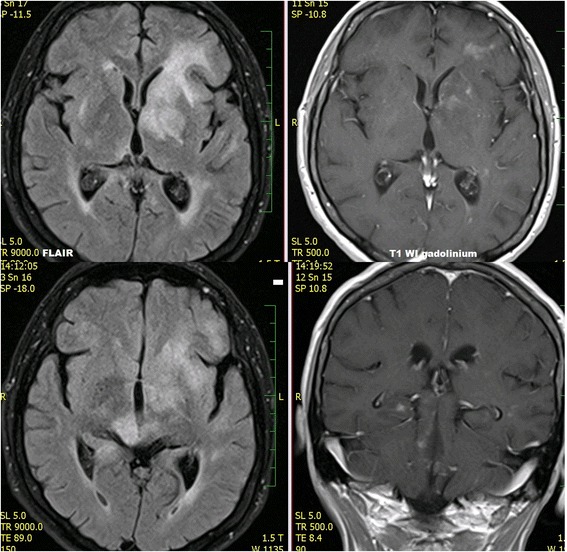
PCNSL. Diffuse infiltrative brain affection

The following signs were assessed on the initial MRI examination (for details, see Table 3): lesion localization; quantity (solitary, multiple and diffuse infiltrative) and quality (demarcated, infiltrative and diffuse infiltrative) of the lesions; type of enhancement and necrosis presence; diffusion restriction presence; meningeal and/or ependymal involvement; cranial nerve involvement; involvement of the corpus callosum, butterfly pattern; involvement of the basal ganglia; presence of perifocal vasogenic edema; signs of bleeding. Inclusion criteria for solitary or multiple infiltrative lesions were as follows (at least one criterion): ill-defined borders; non-enhancing portions of tumor beyond enhancing portion; infiltration of the ependyma, meninges or cranial nerves. Diffuse infiltrative lesions were defined as follows: involvement of both white and grey matter by enhancing tumorous affection (which was nonhomogenous, patchy, worm-like, stripy, etc.) and non-enhancing tumorous infiltration, which was spread along to large white matter tracts and continuously affected at least a) more than 2/3 of one cerebral hemisphere and/or b) different extend of both supratentorial and infratentorial regions. See also Fig. 1. Inclusion criteria for meningeal and ependymal involvement were thickening and enhancing meningeal /ependymal surfaces, which can be smooth, irregular or nodular.

### Statistics

Data were expressed as mean ± SD and as median. Categorical values were analyzed using the chi-squared test. Unpaired t-test was used for the comparison of the time intervals. *P*-values <0.05 were considered significant. Analyses were performed using STATISTICA software version 12.

## Results

### Patient and clinical data

Patient demographic data are summarized in Table 1. In the Table 1 you can also find detailed data about the time intervals from clinical onset to initial brain MRI and initial MRI to intervention (open surgery or stereotactic biopsy). The time interval between clinical onset and initial brain MRI did not significantly differ between PCNSL and GBM; however interval between the initial MRI and stereotactic biopsy/surgery was significantly longer in PCNSL subjects (mean 59 ± 118 days, median 30 days) in comparison to GBM patients (mean 9 ± 7 days, median 8 days), (*p* = 0.002).

**Table 1:** Patient selection data

	PCNSL	GBM
NO of patients	54	54
Sex	26 female, 28 male	21 female, 33 male
Age	62.6 ± 11.5 years (median 65 years)	59 ± 14 years (median 62 years)
Interval from the first clinical manifestation to the first MRI	46 ± 89 days (median 30 days) min. 0 day max. 180 days	49 ± 63 days (median 25 days) min. 0 day max. 270 days
Interval from the first MRI to stereobiopsy or surgery	59 ± 118 days (median 30 days) min. 1 day max. 660 days	9 ± 7 days (median 8 days) min. 1 day max. 30 days

*Max* maximum, *min* minimum, *NO* number

The patients presented with various clinical manifestations (in some patients a combination of multiple manifestations were present; see Table 2). The most common manifestations included organic brain syndrome, signs of intracranial hypertension, paresis, vertigo or phatic disorder. PCNSL patients presented significantly more often with cranial nerve dysfunction and visual disturbance then GBM subjects, (*p* = 0.012) and also in organic brain syndrome, (*p* = 0.038). GBM patients presented significantly more often by seizures then PCNSL patients, (*p* = 0.008).

**Table 2:** Clinical presentation

Clinical symptoms	PCNSL (NO of patients)	GBM (NO of patients)
Organic brain syndrome	22	12
Signs of intracranial hypertension	16	23
Paresis	16	19
Vertigo	12	13
Phatic disorder	11	14
Visual disturbances	7	3
Cranial nerve dysfunction other than visual disturbance	5	0
Fatigue	4	7
Dysesthesia or hypesthesia	4	1
Seizure	1	9

*NO* number

### MRI findings

**Fig. 2:**
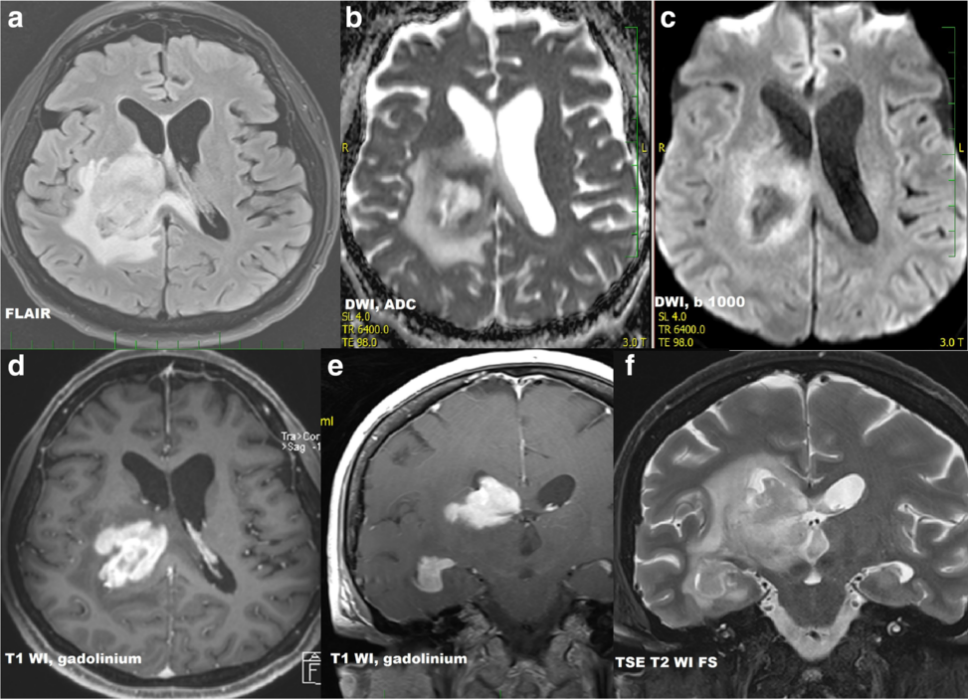
**a**) FLAIR, axial scan **b**) DWI, ADC map, axial scan **c**) DWI, b=1000, axial scan **d**) SE T1 WI after intravenous gadolinium contrast administration, axial scan **e**) SE T1 WI after intravenous gadolinium contrast administration, coronal scan **f**) FSE T2 WI with fat saturation, coronal scan

**Fig. 3:**
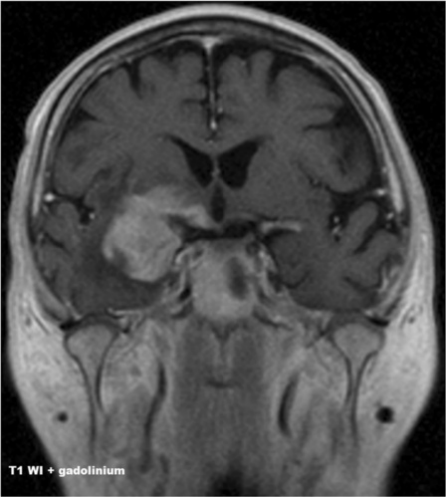
PCNSL. The involvement of the right optic chiasma

**Fig. 4:**
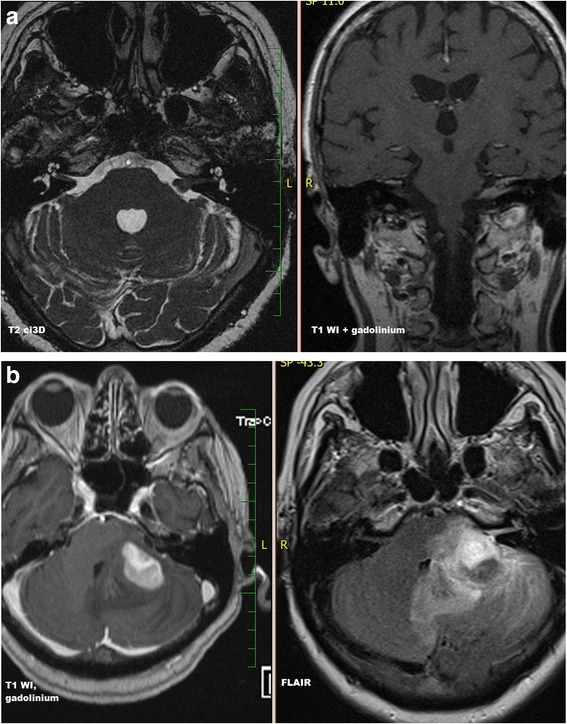
**a** PCNSL. Solitary involvement of the left auditory nerve. **b** PCNSL. Three months follow-up with progression of PCNSL and cerebellar affection

Initial diagnostic MRI results for both groups are summarized in Table 3. PCNSL lesions were generally localized supratentorially (66.7%). PCNSL affected both white and grey matter, basal ganglia involvement was present in 55.6%, and cortical grey matter was affected in 51.9%. Cortical grey matter was involved by both enhancing and non-enhancing tumorous infiltration in 37.1% of cases, only enhancing portion was present in 3.7% of cases, only non-enhancing infiltration was seen in 11.1% of cases. Solitary affection of white matter was found only in 7.4%; in 3.8% an isolated involvement of basal ganglia was present and in one case (1.9%) solitary infiltration of hypothalamus or vestibular nerve was found. At time of initial MRI, PCNSL appeared as multiple infiltrative lesions in 51.9% of cases, as a solitary infiltrative lesion in 20.4% of cases and as a diffuse infiltrative affection in 24.1% (Fig. 1). Solitary demarcated lesions were rare (3.7%). PCNSL enhanced homogenously (64.8%), vasogenic perifocal edema was present in most cases (92.6%), and diffusion restriction was detected in 97% of cases (Fig. 2). Optic nerves and tracts were infiltrated in 42.6% of cases (Fig. 3). Other cranial nerves were affected in 5.6% of PCNSL cases; in one case (1.9%) the optic and trigeminal nerve was affected, and in one case (1.9%) the optic nerves and both auditory and facial nerves were involved and in one case (1.9%) solitary infiltration of the auditory nerve was present without other brain lesions (Fig. 4a, b). PCNSL typically reached the surface of the brain (87%) with meningeal infiltration present in 35.2% of cases and ependymal infiltration in 53.7% of cases. Signs of bleeding (5.6%) were rare.

**Table 3:** MRI findings in PCNSL and GBM at the time of initial evaluation

MRI finding		PCNSL	GBM	*p*-value
Localization	only supratentorial	**66.7%**	**98.1%**	**<0.001**
	only infratentorial	5.5%	1.9%	0.308
	supra- and infratentorial	**27.7%**	**0%**	**<0.001**
Type of lesions	solitary demarcated lesion	**3.7%**	**13%**	**0.046**
	solitary infiltrative type	20.4%	51.9%	0.121
	multiple infiltrative lesions	51.9%	35.2%	0.081
	diffuse infiltrative type	**24.1%**	**0%**	**<0.001**
Type of enhancement	homogeneous	**64.8%**	**0%**	**<0.001**
	nonhomogeneous	**14.8%**	**98.1%**	**<0.001**
	diffuse infiltrative – worm-like, patchy, stripy…	**20.4%**	**0%**	**<0.001**
	presence of necrosis	**5.6%**	**88.9%**	**<0.001**
Involvement of brain surface	without reaching brain surface	12.9%	11.1%	0.567
	reaching brain surface	87%	88.9%	0.846
	meningeal infiltration	35.2%	46.3%	0.240
	ependymal infiltration	53.7%	37%	0.054
Optic nerves, chiasma or tracts involvement		**42.6%**	**5.6%**	**<0.001**
Cranial nerves involvement (optical nerves and tracts excluded)		5.6%	0%	0.079
Corpus callosum infiltration		42.6%	44.4%	0.846
Butterfly pattern		24.1%	14.8%	0.224
Basal ganglia involvement		**55.6%**	**18.5%**	**<0.001**
Cerebral cortex involvement		**83.3%**	**51.9%**	**<0.001**
^a^DWI	free diffusion	1.9%	10.4%	0.189
	restricted diffusion in some part of the solid	97%	89.6%	0.189
Signs of bleeding		**5.6%**	**44.4%**	**<0.001**
Presence of vasogenic edema		92.6%	83.3%	0.139

^a^DWI was available in 35 PCNSL patients and 51 GBM patients. Statisticaly significant results are in bold (*p* less than 0.05)

**Fig. 5:**
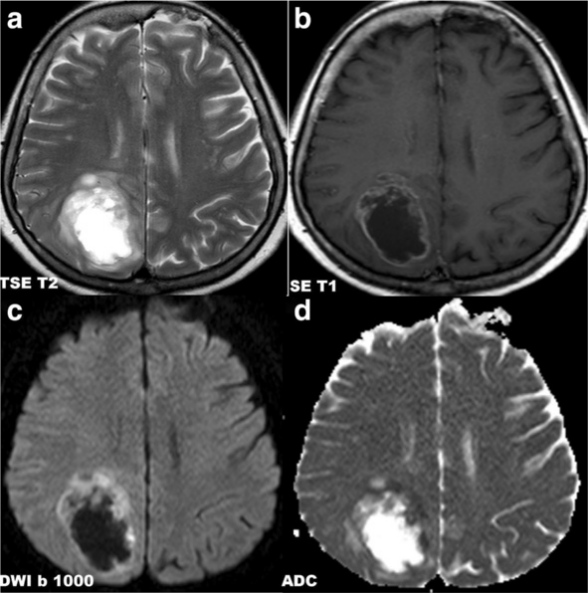
**a**) FSE T2 WI, axial scan **b**) SE T1 WI after intravenous gadolinium contrast administration, axial scan **c**) DWI, b=1000, axial scan **d**) DWI, ADC map, axial scan

**Fig. 6:**
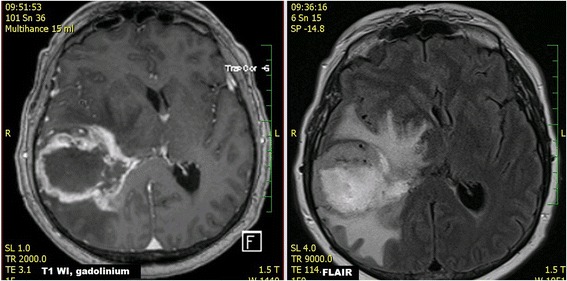
GBM. GBM with ependymal involvement

**Fig. 7:**
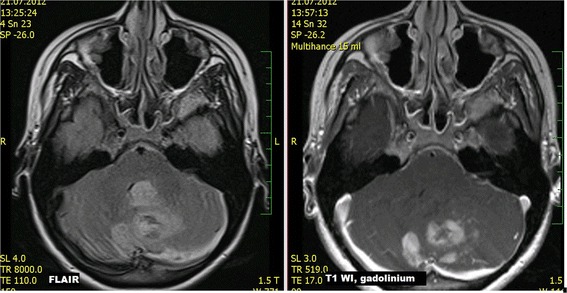
GBM. Cerebellar GBM in a young woman

Nearly all lesions in GBM subjects were localized supratentorially (98.1%); solitary infiltrative lesions were present in 51.9% of cases and multiple lesions in 35.2%. Nonhomogeneous enhancement of GBM lesions was detected in 98.1% of cases and necrosis was present in 88.9% of cases (Fig. 5). GBM dominantly affected white matter; however, in most cases also cortical grey matter was affected (83.3%). In case of cortical involvement by GBM (83.3%), in 63% of cases cortex was affected by enhancing and also non-enhancing portion of tumorous infiltration; in 20.3% of cases cortex was affected only by non-enhancing portion of tumor. Basal ganglia were target in 18.5% and mostly in their margins. Isolated involvement of cortical grey matter was not found. GBM very often reached the surface of the brain (88.9%), and meningeal infiltration was found in 46.3% of cases and ependymal infiltration in 37% of cases (Fig. 6). Vasogenic edema was present in 83.3% of cases. Diffusion restriction was detected in solid portions of the tumor in 89.6% of cases, while no diffusion restriction was observed in the necrotic portion of the tumor. Signs of bleeding were found in 44.4% of cases. In GBM cases optic nerves, chiasma or tracts were infiltrated in 5.6%; the affection of other cranial nerves was not found. Only 2 cases were exceptional. In one case, the lesion was localized infratentorially with nonhomogeneous enhancement (Fig. 7). In the second exceptional case, a non-enhancing supratentorial lesion was present suspected to be low-grade glioma; however, histology confirmed the diagnosis of GBM (WHO Grade IV).

**Fig. 8:**
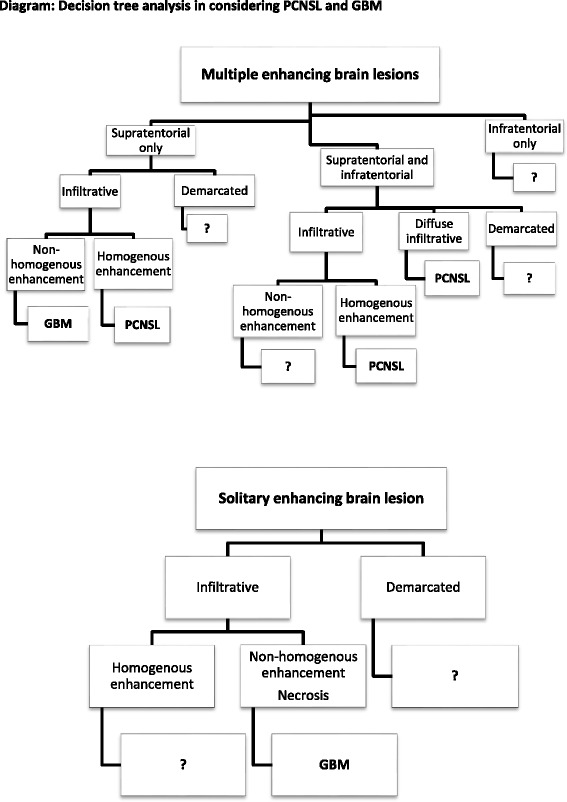
Decision tree analysis in considering PCNSL and GBM

Several significant differences between both brain infiltrative tumors were found (see Table 3). Notably, no homogenous enhancement was found in GBM, in contrast to homogeneous enhancement detected in 64.8% of PCNSL lesions (*p* < 0.001). Enhancement in GBM was nonhomogeneous in 98.1% of cases (no enhancement in 1 case) and necrosis was present in 88.9% of cases. Conversely, necrosis was present only in 5.6% of PCNSL cases and nonhomogeneous enhancement in 14.8% of PCNSL cases (both *p* < 0.001). Diffuse infiltrative type of brain involvement was observed only in PCNSL (24.1% of cases). Additionally, optic pathways infiltration was more frequent in PCNSL than in GBM (*p* < 0.001); present in 42.6% of PCNSL cases and only in 5.6% of GBM cases. Signs of bleeding were more common in GBM (44.4%) than PCNSL (5.6%); *p* < 0.001. Both supratentorial and infratentorial localization was present only in PCNSL (27.7%). The basal ganglia were involved more often in PCNSL (55.6%) than in GBM (18.5%); *p* < 0.001. Finally, cerebral cortex was affected significantly often in GBM (83.3%) than in PCNSL (51.9%); mostly by both enhancing and non-enhancing infiltration. In the Table 4 you can find combinations of several MRI findings and their occurrence in both groups. According those findings we constructed the diagram of the decision tree analysis (Fig. 8). According to Tables 3 and 4, major criteria in decision making process between PCNSL and GBM are the type of enhancement and presence or absence of necrosis. As minor criteria we considered basal ganglia and optic pathways affections, signs of bleeding, both supratentorial and infratentorial localization and diffuse infiltrative type of lesion.

**Table 4:** Combinations of several MRI findings and their occurrence in PCNSL and GBM at the time of their initial evaluation

Combinations of findings	PCNSL	GBM	*p* - value
• Supratentorial • Solitary infiltrative • Non-homogenous enhancement • Necrosis presence	3 (23.1%) of 13 solitary cases (5.6% of all PCNSL)	28 (77.8%) of 36 solitary cases (51.9% of all GBM)	**<0.001**
• Supratentorial • Solitary infiltrative • Non-homogenous enhancement	4 (30.8%) of 13 solitary cases (7.4% of all PCNSL)	34 (94.4%) of 36 solitary cases (63% of all GBM)	**<0.001**
• Multiple infiltrative • Homogenous enhancement	27 (96.4%) of 28 multiple cases (50% of all PCNSL)	0 (0%) of 18 multiple cases (0% of all GBM)	**<0.001**
• Diffuse infiltrative type • No necrosis	13 cases (24.1% of all PCNSL)	0 (0%) of all GBM cases	**<0.001**
• Multiple infiltrative • Non-homogenous enhancement	1(3.6%) of 28 multiple cases (1.9% of all PCNSL)	18 (100%) of 18 multiple cases (33.3% of all GBM)	**<0.001**

Statisticaly significant results are in bold (*p* less than 0.05)

## Discussion

In the present study, we compared morphological MRI characteristics in PCNSL and GBM at time of initial MRI. At initial evaluation, PCNSL lesions were presented as multiple infiltrative lesions, which enhanced homogenously or as diffuse infiltrative affection of the brain. GBM typically manifested as a supratentorial solitary infiltrative tumor nearly in all cases nonhomogeneous enhancement was present with evident necrosis. Both GBM and PCNSL lesions reached the surface of the brain in most cases; meningeal and ependymal infiltration was not uncommon.

We detected several significant differences between PCNSL and GBM lesions. The most striking difference was in enhancement patterns; while homogeneous enhancement was not detected in GBM, most PCNSL lesions (64.8%) enhanced homogeneously. Additionally, necrosis was observed in most GBM lesions (88.9%) but was rare in PCNSL (5.6%). Optic pathways infiltration was common in PCNSL and rare in GBM. Other cranial nerves infiltrations were not frequent and were found only in PCNSL (5.6%). Signs of bleeding were rare in PCNSL and common in GBM. The basal ganglia involvement occurred more frequently in PCNSL than in GBM. Diffuse infiltrative type of brain involvement was observed only in PCNSL (24.1%) and also only PCNSL was localized both supratentorial and infratentorial (27.7%). Finally, cerebral cortex was affected significantly often in GBM (83.3%) than in PCNSL (51.9%); mostly by both enhancing and non-enhancing tumorous infiltration. Solitary non-enhancing tumorous affection of cerebral cortex in both diagnoses was uncommon.

Our MRI findings in GBM are in agreement with those reported previously [17]. However, our findings in PCNSL are only partially consistent with those reported by Haldorsen et al. [18]. In the present study, immunocompetent PCNSL patients presented with multiple lesions in 51.9% of cases, and with involvement of the basal ganglia in 55.6% cases. In contrast, Haldorsen et al. reported multiple lesions in only 35% of PCNSL cases, with basal ganglia involvement in 32% of cases [18]. However, they also reported disseminating lesions in 7% of cases [18]. In our study we used category diffused infiltrative affection and probably this category is equal to Haldorsen´s disseminating lesions. We found diffuse infiltrative brain affection by PCNSL in 24.1% of cases. They also did not report the presence or absence of cranial nerve infiltration [18]. In our study, optic pathways involvement was present in 42.6% of PCNLS cases, other cranial nerves were affected in 5.6%. In one case, solitary auditory nerve involvement was present without other lesions. Several case reports have been published describing solitary involvement of the auditory nerves in PCNSL manifesting by sudden hearing lost [19, 20]. Cranial nerve and leptomeningeal involvement is considered very common in secondary CNS lymphoma [21]; however, systematic lymphoma was excluded in our patients. One difficulty in comparing our results to those reported previously is that many studies are limited by small sample sizes [22, 23]. Therefore, we consider the study of Haldorsen et al. as the most reliable for comparison with our results [18]. Their population-based study evaluated CT/MRI features in 75 AIDS-negative patients in Norway between the years 1989-2003 [18]. However, only 52 patients underwent MRI, the rest of patients were examined only by CT, and considering the fact that sensitivity of CT is significantly lower than MRI, some lesions may have been missed [18]. Haldorsen et al. also included patients with immunosuppression therapy (5%) and also 6 patients, in whom only imaging after corticosteroid therapy was available. Differences between our results are partly explainable by designs of patient selection. We could also a little bit hypothesize about changing imaging findings in the time.

A number of studies have explored advanced MRI techniques such as DWI, perfusion imaging and MR spectroscopy [15, 24]. As PCNSL is highly cellular, diffusion is often restricted. We detected diffusion restriction in 97% of our PCNSL cases. Accordingly, Toh et al. reported significantly lower fractional anisotropy (FA) and apparent diffusion coefficient (ADC) in PCNSL compared to GBM [25]. Although diffusion restriction is also often present in the solid portion of GBM tumors (89.6% in our GBM group), it is important to note than there is generally free diffusion in the necrotic or cystic portions of GBM tumors. The importance of revascularization through angiogenesis for tumor growth has led to a growing interest in novel imaging techniques such as the assessment of tumor vascularity. MR perfusion imaging can visualize nutritive delivery of arterial blood to the capillary bed in tumors. According to published data, PCNSL demonstrates lower relative mean cerebral blood volume than GBM; likely due to massive leakage of contrast media into the interstitial space [15]. In the MR spectroscopy study by Yamasaki et al., a large lipid peak on ^1^H-MR spectroscopy images in PCNSL and small or absent lipid peak in GBM without necrosis was found [24]. MR perfusion and spectroscopic data were available only in few cases in the present study, thus we did not have sufficient data for comparison.

PCNSL and GBM are serious malignant brain tumors with different therapeutic management. Histological verification of PCNSL before oncological treatment is mandatory. Open surgery in PCNSL is not necessary; the diagnostic method of choice is stereotactic biopsy, which is not without complication [26]. We believe that the MRI morphological differences between PCNSL and GBM reported in the present study may be useful in daily radiological practice and may help to differentiate between both malignant entities.

The present study has several limitations. Due to its retrospective nature, some MRI examinations were of lower quality and acquired on different whole-body systems. As stated above, we did not have sufficient data for reviewing advanced MRI techniques such as MR spectroscopy or perfusion, also we do not have sufficient data for FA evaluation and not all patients underwent DWI and for measurement of ADC value. In limitations of the study we must mention the fact of relative incidence of both conditions. PCNSL occur less frequently than GBM. This fact makes the diagnosis more complex as no simple morphological feature is able to discriminate between these conditions. We tried to extract combinations of relevant features and provide radiological clues for further work-up.

## Conclusions

Routine morphological MRI is capable of differentiating between GBM and PCNSL lesions in many cases at time of initial presentation. PCNSL often presented as multiple lesions that enhanced homogenously, often with optic pathways infiltration and involvement of the basal ganglia or as diffuse infiltrative type of brain involvement. A solitary supratentorial lesion with nonhomogeneous enhancement and necrosis presence was typical for GBM.

## Abbreviations

ADC: apparent diffusion coefficient
CNS: central nervous system
DLBCL: diffuse large B-cell lymphoma
DWI: diffusion-weighted images
FLAIR: T2-weighted fluid-attenuated inversion recovery
FSE: fast spin echo
GBM: glioblastoma
GE: gradient echo
MRI: magnetic resonance imaging
PCNSL: primary central nervous system lymphoma
SWI: susceptibility-weighted images
T: tesla
T1 WI: T1 weighted images
T2 WI: weighted images
